# Particulate Blood Analogues Reproducing the Erythrocytes Cell-Free Layer in a Microfluidic Device Containing a Hyperbolic Contraction

**DOI:** 10.3390/mi7010004

**Published:** 2015-12-30

**Authors:** Joana Calejo, Diana Pinho, Francisco J. Galindo-Rosales, Rui Lima, Laura Campo-Deaño

**Affiliations:** 1 ESTiG, Polytechnic Institute of Bragança, C. Sta Apolónia, 5301-857 Bragança, Portugal; jo_calejo6@hotmail.com (J.C.); diana@ipb.pt (D.P.); 2 CEFT, Faculty of Engineering of the University of Porto, Rua Dr. Roberto Frias, 4200-465 Porto, Portugal; galindo@fe.up.pt; 3 MEtRiCS, Mechanical Engineering Department, University of Minho, Campus de Azurém, 4800-058 Guimarães, Portugal; rl@dem.uminho.pt

**Keywords:** hemodynamics, blood analogue, rheology, microfluidics, cell-free layer

## Abstract

The interest in the development of blood analogues has been increasing recently as a consequence of the increment in the number of experimental hemodynamic studies and the difficulties associated with the manipulation of real blood *in vitro* because of ethical, economical or hazardous issues. Although one-phase Newtonian and non-Newtonian blood analogues can be found in the literature, there are very few studies related to the use of particulate solutions in which the particles mimic the behaviour of the red blood cells (RBCs) or erythrocytes. One of the most relevant effects related with the behaviour of the erythrocytes is a cell-free layer (CFL) formation, which consists in the migration of the RBCs towards the center of the vessel forming a cell depleted plasma region near the vessel walls, which is known to happen in *in vitro* microcirculatory environments. Recent studies have shown that the CFL enhancement is possible with an insertion of contraction and expansion region in a straight microchannel. These effects are useful for cell manipulation or sorting in lab-on-chip studies. In this experimental study we present particulate Newtonian and non-Newtonian solutions which resulted in a rheological blood analogue able to form a CFL, downstream of a microfluidic hyperbolic contraction, in a similar way of the one formed by healthy RBCs.

## 1. Introduction

Understanding the hemodynamics and the fluid-structure interactions of the blood flowing through the microvasculature system is of great importance, since it is related with the correct functionality of the material transport and exchange [1]. The complex flow behaviour of blood is closely associated with the main cardiovascular diseases and for these reasons several numerical and experimental (*in vitro* and *in vivo*) studies have been carried out during the last decades [2,3,4,5,6,7,8,9,10]. In these studies Newtonian and non-Newtonian approaches for the blood flow were taken into account. Nevertheless, none of these approaches takes into consideration the role of the individual red blood cells (RBCs) or erythrocytes, which are the predominant component of blood, with a contribution of around 45% by volume. Under normal flow conditions the hemodynamics are dominated by the interaction of the RBCs with the vessel walls and the plasma, which is essentially water made, but slightly more viscous and dense because of the presence of dissolved proteins. Therefore, the viscosity of blood depends on different factors such as the RBCs concentration, flow rate and the vessel diameter [11]. This latter factor is one of the most important, since an axial migration of the deformable RBCs in the microvessels is observed when the diameter of the vessel diminishes below approximately 300 µm [12], which leads to the lowering of the apparent viscosity of blood [13]. This effect is known as the Fåhræus-Lindqvist effect [14] which gives place to a cell-free layer (CFL) of plasma.

Several studies for CFL measurement exist in both *in vitro* [15,16,17,18,19] and *in vivo* [20,21,22] environments. Most of the *in vitro* studies were carried out using simple straight microchannels. However, recently more complex geometries such as bifurcation and confluence have been used. For example, Ishikawa *et al.* [23] and Leble *et al.* [24] have demonstrated the existence of a thin CFL in the center of the microchannel just downstream of a confluence. The development of the microscopic technology and new materials suitable for microchannel fabrication have made possible to study the fluid dynamics within the microchannel. Hence, the enhancement of CFL thickness has been examined in microchannels with a sudden contraction [25,26] and a hyperbolic shaped contraction [27,28] followed by a sudden expansion plane. This phenomenon was applied to a complete extraction of blood cells [25,29] and partial separation of RBCs [30,31] from plasma. Most of these studies were developed using human blood diluted with dextran 40 or physiological saline [15,17,18,19,24,30,32] in order to achieve the microcirculation hematocrit (∼25%), or using RBCs of other mammals such sheeps [33,34], that demonstrated have close behaviour to the human RBCs. Despite these experimental studies, as the manipulation of real blood is often difficult due to the ethical, economical and safety issues involved, the development of blood analogue solutions is needed. In this sense, the literature compiles different one-phase solutions with both Newtonian and non-Newtonian characteristics able to mimic the rheological properties of real blood [8,35,36,37,38]. Nevertheless, it would be rather more interesting having a particulate-viscoelastic blood analogue able to mimic simultaneously both the rheological behaviour of real blood as a whole, and the physiological response of the RBCs represented by the dispersed particles in the solution. Until now, very few works related with particulate blood analogues have been carried out. Maruyama *et al.* [39,40] developed microcapsule suspensions in a Newtonian solvent to evaluate the absolute hemolytic properties of centrifugal blood pumps. Later on Nguyen *et al.* [41] elaborated similar blood analogues for the study of hemolysis using in this case a non-Newtonian solvent. However, even when these blood analogues are able to reproduce the rheological properties of blood, none of them has focused on CFL formation and enhancement. In this work we have developed particulate Newtonian and non-Newtonian solutions made of dextran and xanthan gum with rigid PMMA (polymethylmethacrylate) spherical particles able to mimic simultaneously both the rheological properties of RBCs in dextran and the effect of CFL formation that frequently happens in *in vitro* blood flow systems. A rheological characterization under shear flow was carried out in order to obtain the viscosity curves under steady state. Flow visualizations through hyperbolic contraction microchannels made of PDMS by means of soft lithography were used to observe the CFL originated by the blood analogues flowing through the microchannel and then compared with the cell-free layer formed by RBCs.

## 2. Experimental Section

### 2.1. Working Fluids and Microchannel Geometry

In this study we have used different working fluids which are summarized in Table 1, their composition is as follows:

- Dextran 40 (Dx40, $\rho_{\text{Dx40}}$ = 1.05 g/cm${}^{3}$), containing 5% by volume of ovine RBCs, *i.e.*, hematocrit of 5% (*v/v*);
- A two-phase viscoelastic solution made of xanthan gum (115 ppm) diluted in the solvent dextran 40, carrying 5% by weight (4.4% *v/v*) of PMMA spherical particles of 6 µm diameter ($\rho_{\text{PMMA}}$ = 1.20 g/cm${}^{3}$) and 0.05% by weight of sodium dodecyl sulfate (SDS);
- Dextran 40 carrying 5% by weight (4.4% *v/v*) of PMMA spherical particles of 6 µm diameter and 0.05% by weight of sodium dodecyl sulfate (SDS);
- Additionally, a solution of only Dx40 and another solution of xanthan gum (115 ppm) diluted in Dx40 were also analyzed.

**Table 1:** Composition of the working fluids.

Acronym	PMMA	XG	Dx40	RBC’s
*X1*	-	115 ppm	✓	-
*X2*	5% (*w/w*)	115 ppm	✓	-
*D1*	-	-	✓	-
*D2*	5% (*w/w*)	-	✓	-
*B1*	-	-	✓	5% (*w/w*)

For the preparation of the particulate-viscoelastic solution, the xanthan gum was mixed with Dx40 by means of a small magnetic stirrer at low speeds in order to prevent mechanical degradation of the polymer molecules during 3 days at room temperature until the solution becomes clear; afterwards the PMMA 6 µm diameter microparticles and the SDS were added. The SDS is added in order to avoid microparticles aggregation. On the other hand, the RBCs solution consisted of ovine RBCs dispersed in a Dx40 at 5% by volume. The blood was collected from a healthy adult ovine, and ethylenediaminetetraacetic acid (EDTA) was added to prevent coagulation. The RBCs were separated from the plasma and buffy coat by centrifugation (2500 rpm for 10 min). The RBCs were then washed and centrifuged with physiological saline twice. Washed RBCs were diluted with Dx40 to make several samples with hematocrit levels of 5% (*v/v*). All blood samples were stored hermetically at 4 ${}^{\circ }C$ until the labelling. It is important to highlight that we used ovine RBCs suspended in Dx40 with dimensions ∼5 µm [42], close to the dimensions of the PMMA particles (6 µm). Human and sheep RBCs present common characteristics, since both species are mammals. Both RBCs are not nucleated, have biconcave disk shape and the function of transporting haemoglobin. Also it has already observed by some authors that ovine RBCs suspended in Dx40 show migration to the center line and consequent CFL formation [33] and deformation through a hyperbolic geometry [34], similarly to human RBCs, when flowing through a microchannel.

**Figure 1:**
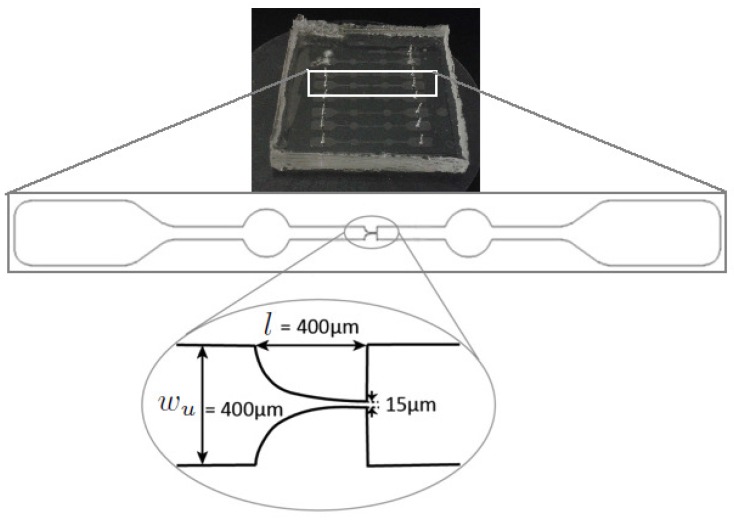
A schematic top-view of the microchannel. The width ($w_{u}$) and contraction length (*l*) are 400 µm. The width of the exit of the contraction region ($w_{c}$) and the depth of the channel (*h*) were 15 µm.

**Figure 2:**
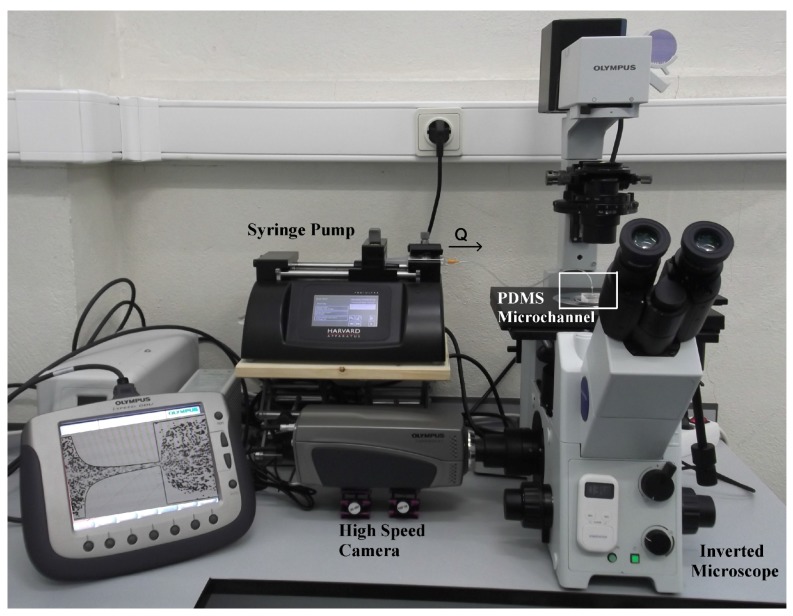
A view of experimental set-up: an inverted microscope (IX71, Olympus) and a 10× objective lens, combined with a high-speed camera (i-SPEED LT, Olympus). The PDMS microchannel was placed on the stage of the microscope where the flow rate (*Q*) of the working fluids was kept constant (at 5 and 20 µL/min) by means of a syringe pump (Harvard Apparatus PHD ULTRA).

Polydimethylsiloxane (PDMS) microchannels were fabricated by using a soft-lithography technique. These microchannels were designed with a hyperbolic contraction followed by a sudden expansion, which is a well-defined geometry for the analysis of contraction-expansion flows in microfluidics, due to its ability to generate strong extensional flows with homogeneous strain-rate near the centerline [38,43]. As described in Figure 1, the dimensions of the microchannel are given by 400 µm ($w_{u}$) × 400 µm (*l*) × 15 µm ($w_{c}$) where $w_{u}$, *l* and $w_{c}$ refer to the width of the inlet microchannel, the length of the hyperbolic contraction region and the width of the contraction, respectively. Thus the total Hencky strain, defined as $\epsilon_{H}=ln\frac{w_{u}}{w_{c}}$, was 3.3. Moreover, the depth (*h*) of the planar microchannel was kept constant at 15 µm. The high-speed video microscopy system used in the present study consists of an inverted microscope (IX71, Olympus, Tokyo, Japan) and a 10× objective lens with a numerical aperture of 0.25, combined with a high-speed camera (i-SPEED LT, Olympus). The PDMS microchannel was placed on the stage of the microscope where the flow rate (*Q*) of the working fluids was kept constant (5 and 20 µL/min) by means of a syringe pump (Harvard Apparatus PHD ULTRA, Holliston, MA, USA) with a 1 mL syringe (TERUMO^®^ SYRING). These flow rates were chosen based on the typical value of mean velocity of human blood in small vessels, ∼3 cm/s [44]. An illustration of the experimental set-up is shown in Figure 2. The images of the flowing RBCs/PMMA particles were captured at the mid-plane of the micro-channel using the high speed camera at 800 fps and were then transferred to the computer to be analyzed.

### 2.2. Fluid Rheology

The rheological characterization of the working fluids were carried out by means of a stress control rheometer (Physica MCR-301, Anton Paar, Graz, Austria), using a 50 mm diameter plate-plate geometry with a gap of 0.1 mm. Although the plate-plate geometry does not ensure a constant shear rate through the whole volume of the fluid sample, secondary flows appear at larger values of shear rate, which allowed us to obtain suitable steady viscosity curves from 1 to 10,000 s${}^{-1}$. In the case of the solution *B1*, the rheological measurements were carried out using another stress control rheometer (Bohlin CVO, Malvern, Worcestershire, UK) equiped with a cone-plate geometry of 60 mm diameter and a angle of 1${}^{\circ }$. At least three replicates with fresh samples in each measurement were made in order to corroborate the reproducibility. All measurements were carried out at $20{\;}^{\circ }$C.

### 2.3. Image Analysis

**Figure 3:**
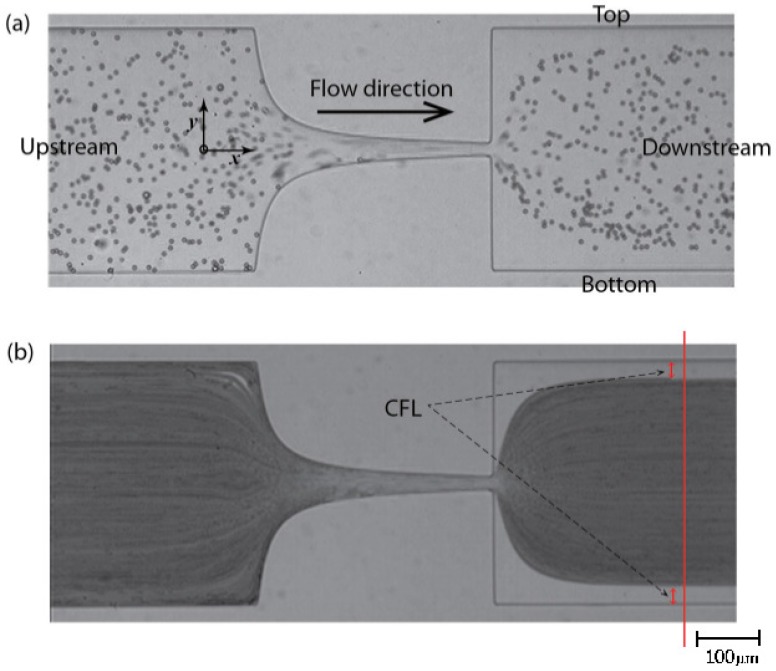
(**a**) An original image of polymethylmethacrylate (PMMA) particle flow and (**b**) its minimum intensity image; the vertical line is the measuring position placed at 300 µm from the contraction exit.

In order to measure the enhancement of CFL downstream of the microchannel (*cf*. Figure 3a), the video sequences captured by a high-speed camera were digitally processed using an image handling software ImageJ [28,45]. First, the AVI video files were converted to the sequences of JPEG image files so that each static image corresponds to a single video frame. The size of the image was 800 pixels wide and 600 pixels high. Basically, intensity level distinction was used to identify the CFLs since it is relatively clear that RBCs and PMMA particles are represented as dark (low intensity) circular shapes against bright (high intensity) background in the images. Therefore, the minimum intensity level was selected from all the images at each pixel position and only one image replaced with these minimum intensity values was created by means of “Z Project” function in ImageJ. The resulted image can be seen in Figure 3b. In this image, the CFLs are identified as high intensity regions between microchannel walls and RBCs/PMMA particles concentration region both shown as low intensity areas. The distance between the wall and RBCs/PMMA particles core was measured at both sides, named as top and bottom in Figure 3a, and averaged for the mean width of CFL. The measuring position was equally set for all the cases 300 µm downstream in *x*-direction from the contraction exit. More than five videos were analyzed in the same way for each case of fluid sample in order to ensure the reliability of the results.

## 3. Results and Discussion

### 3.1. Results

**Figure 4:**
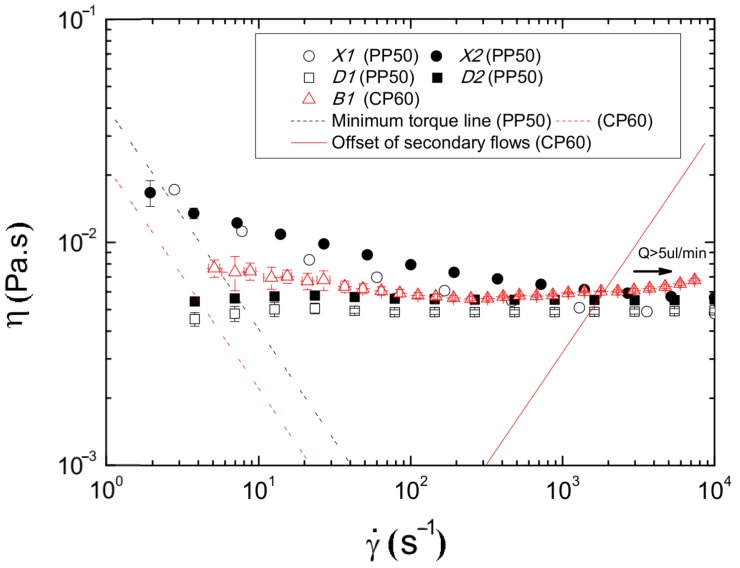
Steady shear viscosity curves for the viscoelastic solution with and without PMMA particles and for the Dx40 solution. The minimum torque line represents the limit of accuracy of the rheometer.

The flow behaviour of the different samples (*X1*, *X2*, *D1*, *D2* and *B1*) under steady state experiments is shown in Figure 4. Two tendencies in their viscosity curves are clearly distinguished. On one hand, samples *D1* and *D2* present a clear Newtonian flow behaviour, with a constant viscosity of 4.9 and 5.5 mPa·s, respectively. On the other hand, the shear viscosity of samples *X1* and *X2* decreases as the shear rate increases (shear thinning behaviour) until a certain value of shear rate (∼500 s${}^{-1}$) at which the viscosity remained constant at similar values as *D1* and *D2*.

In relation with the Newtonian samples, the differences in their viscosity values are due to the addition of the PMMA particles, this effect is supported by means of the Einstein’s modified equation [46,47], in which the increment of the viscosity is directly proportional to the concentration of rigid spherical particles. The same effect is observed for the solutions *X1* and *X2*, that apart from the increased values of viscosity (13.26%), the addition of 5% *w/w* of PMMA particles slightly reduces the degree of shear-thinning. Moreover, as it has been reported by Campo-Deaño *et al.* [8], solutions with 100 ppm of xanthan gum have certain degree of elasticity with a relaxation time close to 2 ms which allows one to consider these samples as viscoelastic solutions.

In the middle of these two observed tendencies we can find sample *B1*, at low shear rates presents a slightly shear thinning behaviour with a constant viscosity at high shear rates. However, no sign of elasticity has been observed for this sample.

Thus, samples *D2* and *X2* presents a viscosity behaviour able to mimic the viscosity of real RBCs in dextran [48,49] at high shear rates. In order to corroborate that these particulate-viscoelastic blood analogues can be also considered as simplified physiological blood analogues able to form a CFL downstream from the contraction in the microchannel as RBCs do, the CFL formed in the hyperbolic contraction microchannel by samples *X2* and *D2* are compared with sample *B1*.

**Figure 5:**
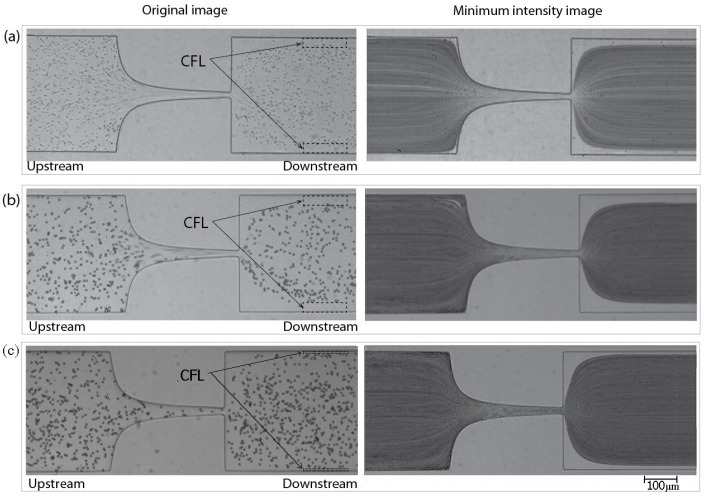
Images of (**a**) Red Blood Cells (RBCs) flow, *B1*, (**b**) two-phase viscoelastic solution flow, *X2* and (**c**) PMMA particles in Dx40 flow, *D2*. **Left**: an original image; **Right**: a minimum intensity image.

As can be seen in Figure 5, CFL formation was observed for sample containning the ovine RBCs (*B1*) and for samples containing PMMA microparticles (*X2* and *D2*), more clearly downstream from the contraction region, whereas upstream the CFL was negligible. The mean thicknesses of the CFLs are compared for the two particulate solutions downstream the contraction and for the RBCs suspended in Dx40.

### 3.2. Discussion

**Figure 6:**
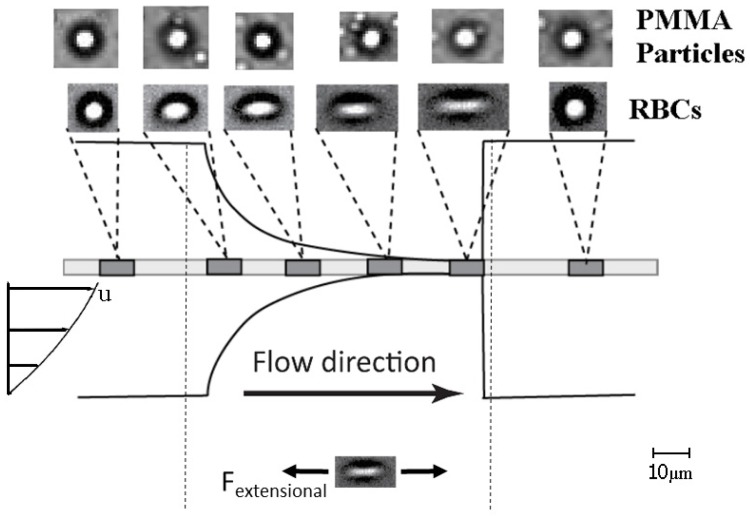
Illustration of the RBCs deformability through hyperbolic microchannel where F${}_{\text{extensional}}$ represents the strong extensional force applied to the RBCs. The same forces was applied to the PMMA particles however without present any deformation.

The hyperbolic shape of the contraction and the subsequent generated extensional flow enlarged the CFL of both fluids. The flow in this geometry along the center line of the channel linearly increases the local velocity, maintaining at the same time the strain rate approximately constant. RBCs flowing through hyperbolic geometries and due to their membrane properties present large deformability under strong extensional flow generated in the middle of the microchannel which is dominant over the shear flow, as is illustrated in Figure 6. As a result, the RBCs tend to elongate as they flow through the contraction. Yaginuma *et al.* [28] found that RBCs change from a nearly circular to an elliptical shape and become increasingly elongated as they flow through a hyperbolic contraction. In the case of rigid particles, Pinho *et al.* [30] observed that the polystyrene (PS) particles of 10 µm flowing through a pronounced contraction present a very small deformation in comparison to ovine RBCs.

**Figure 7:**
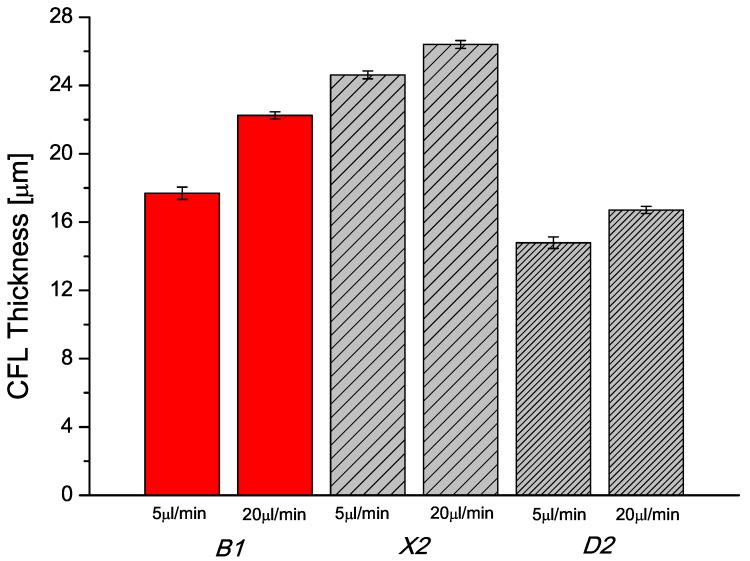
Comparison of mean CFL thickness in downstream region between the fluid *B1*, RBCs suspended in Dx40; *X2*, particulate-viscoelastic fluid and *D2*, PMMA particles in Dx40, as a function of flow rate, 5 µL/min and 20 µL/min. The error bar means 95% confidence interval.

RBCs, due to their highly flexible structure and the parachute effect, tend to concentrate towards the centre of the channel pulled by the flow. Meanwhile, PMMA particles in sample *D2* as they are rigid spheres, the opposite behaviour is expected. This has been corroborate in Figure 7 by the PMMA particles dispersed in a Newtonian carrier fluid, which formed a smaller CFL thickness after the contraction region compared to that formed by the RBCs. In opposition, the PMMA particles dispersed in a non-Newtonian carrier fluid formed a larger CFL after the contraction region, about 8 µm larger than RBCs. This is an interesting phenomena that some researchers have already observed and study [50,51,52,53]. The explanation for this deviation has to be found in the rheological differences existing for the suspending fluids. On the RBCs suspension, the carrier fluid is Newtonian (no elasticity and constant viscosity), while in the case of sample *X2* the PMMA particles are suspended in a shear thinning and slightly elastic fluid. At the shear rates corresponding to applied flow rates in the microchannel, the viscosity behaviour for all the sample is Newtonian (see Figure 4), however in the case of sample *X2* the carrier fluid presents certain degree of elasticity given by the amount of polymer in the solution. This elasticity seems to be the responsible for this particle migration towards the center of the microchannel, opposite to the effect with the RBCs and PMMA suspended in the Newtonian solvent. Non-Newtonian effects provide a mechanism for the cross-stream particle migration in microfluidic confined channels, which has triggered several investigations in recent years. This phenomenon was observed experimentally by several researchers [50,51,52,53], and they have observed different particle migration directions depending on the properties of the polymeric fluid. For instance, rigid spheres tend to migrate laterally towards the wall in high shear-thinning fluids while are attracted towards the centre line in elastic and smooth shear-thinning fluids [52]. Additionally, Tehrani [54] found that in a fluid with suspensions of 5%–12% particle volume fraction (such as the tested fluids with ∼5% of PMMA particles), the migration of the particles tend to occur towards the region of lower shear rate, *i.e.*, to the centre line of the microchannel [52]. For the tested sample *X2*, ∼5% of particles were suspended in a fluid with some viscoelastic characteristics. The viscoelastic properties of this fluid will proportionate a smooth shear thinning behaviour of the carrier fluid (as shown in Figure 4) that can be responsible for the string formation of particles by contributing in the lubrication forces [55]. This would be a possible explanation for the observed differences in the CFL thickness downstream of the contraction region. Nevertheless, in our study we have used a Reynolds number always bigger than 1 and as a result the shear thinning effect should be negligible at such high flow rates. Hence, in this particular study, the major responsible of the observed differences is more likely to be due to the normal stress difference, which also contributes to the CFL enhancement . In order to clarify this phenomenon, further investigation with different geometries and Non-Newtonian fluids is currently under way and the results will be shared in due course.

Figure 7 also shows that the CFL thickness is slightly affected by the flow rate and two different behaviors were observed. On one hand, at 5 µL/min sample *D2* presents a CFL more similar to the one observed in *B1*. On one hand, at 5 µL/min sample *D2* presents a CFL more similar to the one observed at the sample *B1*. On the other hand, at higher flow rates (20 µL/min), more close CFL thickness was found between sample *X2* and *B1*. This could be explained by the fact that the extensional effect of the RBCs is largely promoted by the increment of the flow rate, and subsequently increasing the CFL at 20 µL/min. In the case of the rigid particles, the increment of the flow rate increases the CFL in the same degree, regardless the elasticity of the carrier fluid. Additionally, it can be observed that the CFL differences between 5 and 20 µL/min of each proposed blood analogue is associated with the small inertia effects. The tested flow rates are out of the Stoke flow conditions (Re ≪ 1), since the Reynolds numbers in our study ranges from ∼1.2 to ∼4.7.

## 4. Conclusions

In this study, simplified particulate Newtonian and non-Newtonian solutions able to mimic the viscosity behaviour of RBCs in dextran at high shear rates were successfully developed. Both solutions were based on dextran 40 with rigid spheres particles of PMMA. One of them exhibited viscoelastic behaviour due to the addition of xanthan gum. Moreover, it was corroborated that the PMMA particles had different tendencies to migrate towards the microchannel centre depending on the rheological properties of the carrier fluid. In spite of the fact of using rigid particles, the CFL in both cases resembles the CFL thickness formed by real RBCs suspended in dextran 40 (∼5%). However, the use of rigid particles limits the physiological realism of the current study and, therefore, future works are directed towards the development of particulate-viscoelastic blood analogues based on deformable particles.
